# Clinical Efficacy of Doxycycline for Treatment of Macrolide-Resistant *Mycoplasma pneumoniae* Pneumonia in Children

**DOI:** 10.3390/antibiotics10020192

**Published:** 2021-02-17

**Authors:** Hyunju Lee, Youn Young Choi, Young Joo Sohn, Ye Kyung Kim, Mi Seon Han, Ki Wook Yun, Kyungmin Kim, Ji Young Park, Jae Hong Choi, Eun Young Cho, Eun Hwa Choi

**Affiliations:** 1 Department of Pediatrics, Seoul National University College of Medicine, Seoul 03080, Korea; hyunjulee@snu.ac.kr (H.L.); pedwilly@snu.ac.kr (K.W.Y.); 2 Department of Pediatrics, Seoul National University Bundang Hospital, Seongnam 13620, Korea; kyungman1126@gmail.com; 3 Department of Pediatrics, Seoul National University Children’s Hospital, Seoul 03080, Korea; cyypedr@gmail.com (Y.Y.C.); tadd17@hanmail.net (Y.J.S.); blossom0225@naver.com (Y.K.K.); 4 Department of Pediatrics, Seoul Metropolitan Government-Seoul National University Boramae Medical Center, Seoul 07061, Korea; msh-0827@hanmail.net; 5 Department of Pediatrics, Chung-Ang University Hospital, Seoul 06973, Korea; ji8303@gmail.com; 6 Department of Pediatrics, Jeju National University School of Medicine, Jeju 63241, Korea; ongsyunju@gmail.com; 7 Department of Pediatrics, Chungnam National University Hospital, Daejeon 35015, Korea; pedeyc@gmail.com

**Keywords:** *Mycoplasma pneumoniae*, macrolides, drug resistance, doxycycline

## Abstract

In areas with high prevalence of macrolide-resistant *Mycoplasma pneumoniae* (MRMP) pneumonia, treatment in children has become challenging. This study aimed to analyze the efficacy of macrolides and doxycycline with regard to the presence of macrolide resistance. We analyzed children with MP pneumonia during the two recent epidemics of 2014–2015 and 2019–2020 from four hospitals in Korea. Nasopharyngeal samples were obtained from children with pneumonia for MP cultures and polymerase chain reaction (PCR). Macrolide resistance was determined by the analysis of 23S rRNA gene transition. Time to defervescence and to chest X-ray improvement were analyzed. Of 145 cases, the median age was 5.0 years and MRMP accounted for 59 (40.7%). Among macrolide-susceptible MP (MSMP), 78 (90.7%) were treated with macrolides and 21 (35.6%) in the MRMP group with doxycycline. In MRMP pneumonia, shorter days to defervescence (2 vs. 5 days, *p* < 0.001) and to chest X-ray improvement (3 vs. 6 days, *p* < 0.001) in the doxycycline group than in the macrolide group was observed, whereas no differences were observed among children with MSMP pneumonia. Compared to macrolides, treatment with doxycycline resulted in better outcomes with a shorter time to defervescence and to chest X-ray improvement among children with MRMP pneumonia.

## 1. Introduction

*Mycoplasma pneumoniae* (MP) is an important cause for community-acquired pneumonia in children and adults [1,2]. MP infections in children are known as mild and self-limiting; however, antimicrobial treatment is recommended in children with moderate to severe lower respiratory tract infections [1,3,4].

MP is distinguished from other bacteria due to unique microbiologic characteristics, including lack of a cell wall, which attributes to being intrinsically resistant towards beta-lactams [5]. Therefore, antibiotics that act on the bacterial ribosome and inhibit protein synthesis such as macrolides or tetracyclines or agents that inhibit DNA replication such as fluoroquinolones are active against MP [6,7]. Among these, macrolides are recommended as first-line treatment for MP pneumonia in children due to the potential toxicities of other agents in children. However, in cases with delay in defervescence and clinical deterioration, macrolide-resistance should be considered. Delayed effective treatment is associated with prolonged and/or more severe presentation [8]. Macrolide-resistance has emerged worldwide with a relatively high prevalence in Asia, ranging from 23.3% to over 84.8% [7]. Therefore, treatment with the increase in macrolide-resistance has become challenging.

In this study, we aimed to compare the efficacy of macrolides and doxycycline in children with MP pneumonia treated in the context of no information regarding macrolide resistance, which were retrospectively tested and classified as macrolide-sensitive MP (MSMP) and macrolide-resistant MP (MRMP).

## 2. Results

### 2.1. Patient Characteristics

**Figure 1:**
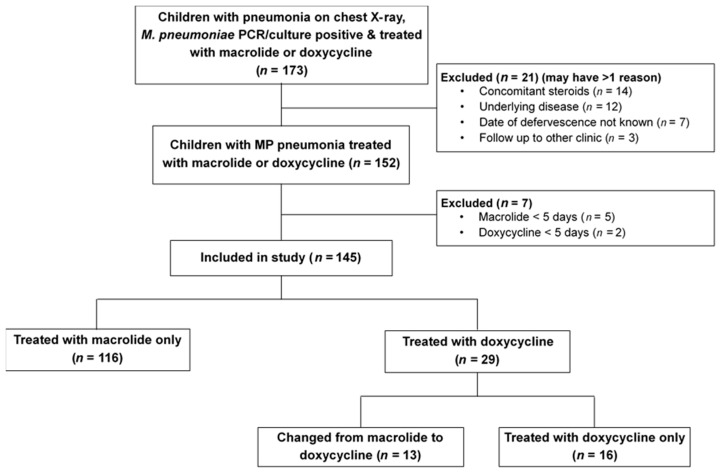
Flow diagram of cases included in the study for comparison of antibiotic response between macrolides and doxycycline in children with *Mycoplasma pneumoniae* pneumonia according to macrolide resistance. MP, *Mycoplasma pneumoniae*.

During the two epidemics of 2014–2015 and 2019–2020, 173 cases were positive for MP PCR or culture and diagnosed with MP pneumonia. Among 173 cases, 21 cases were excluded due to at least one of the following: concomitant steroids (*n* = 14), underlying disease (*n* = 12), date of last fever not specified (*n* = 7), and follow up to other institution (*n* = 3) (Figure 1). Additionally, children treated for less than 5 days (macrolide *n* = 5, doxycycline *n* = 2) were also excluded. Finally, 145 cases were included in the analysis; 103 (71.0%) during the 2014–2015 season and 42 cases (29.0%) from the 2019–2020 season.

The demographics and clinical characteristics of cases included in this study are shown in Table 1. Among 145 cases, 59 (40.7%) were male and the median age was 5.0 years. There was no difference of median age between MRMP and MSMP groups. Eight cases (5.5%) had underlying diseases (neurologic disease, *n* = 1; hematologic disease, *n* = 3; cardiac disease, *n* = 1; endocrine disease, *n* = 1; gastrointestinal disease, *n* = 2). Among the cases tested, 92.0% (127/138) were MP PCR positive, 62.7% (32/51) were anti-mycoplasma IgM positive, 48.3% (57/118) showed anti-mycoplasma IgG ≥ 1:640, and 94.6% (35/37) showed a four-fold increase. The median duration of fever was 7 days and children in the MRMP pneumonia group had significantly longer duration of fever (8 days vs. 7 days, *p* value = 0.003). Median duration of cough was 15 days and there was no significant difference according to macrolide resistance. More than one-third of the cases showed lobar consolidation and 11.0% had parapneumonic effusion. There was no difference in radiologic findings according to macrolide susceptibility. Among the cases, 12.4% had combined viral co-infection with a similar distribution between MSMP and MRMP groups. Oxygen therapy was required in 2.8% and there were no cases on mechanical ventilator. Two cases had complications including bronchiolitis obliterans and persistent large-scale atelectasis. 

### 2.2. Macrolide Resistance and Clinical Efficacy

Cases of MRMP accounted for 40.7% (*n* = 59) in this study (Table 1). Macrolide resistance rate differed between institutions. Institutions in Seoul, Seongnam, and Daejeon showed macrolide resistance of 74.1%, 63.6%, and 66.7%, respectively. In contrast, macrolide resistance from the institution on Jeju Island was 10.8%. Macrolide resistance was 30.1% in the 2014–2015 season and 66.7% in the 2019–2020 season; the difference between the two seasons is related to the distribution in cases included according to district.

Among all cases, 116 (80.0%) were treated with macrolides only, 13 (9.0%) changed from macrolide to doxycycline, and 16 (11.0%) were treated with doxycycline only (Table 2). In the MSMP group, 78 (90.7%) were treated with macrolides; in the MRMP group, 21 (35.6%) were treated with doxycycline. According to the antibiotic regimen, children treated with doxycycline (including those changed from macrolides) were older than children treated with macrolides only (Table S1). The macrolide resistance rate was also higher in children initially treated with macrolides changed to doxycycline (11/13, 84.6%) and doxycycline only (10/16, 62.5%) compared with children treated with macrolide only (38/116, 32.8%) (*p* < 0.001) (Table S1).

Among children treated with macrolides, clarithromycin (*n* = 53) was most commonly prescribed, followed by roxithromycin (*n* = 42) and azithromycin (*n* = 6), and cases which changed the macrolide agent during therapy (*n* = 28) were also included in the study. The median duration of antibiotic use for macrolides and doxycycline were both 10 days, respectively. Among the 13 cases in which macrolide was changed to doxycycline, data on macrolide initiation date was available for 11 cases and the median days to change to doxycycline was 4 days.

In the analysis of clinical response according to macrolide resistance, the duration of fever and days to chest X-ray improvement after initiation of macrolides was shorter in MSMP compared with MRMP. There was no difference according to macrolide resistance in duration of fever or days to chest X-ray improvement in those treated with doxycycline (Table 3).

When comparing clinical response between antibiotics, among MSMP there was no difference in duration of fever or days to chest X-ray improvement in the macrolide group compared with the doxycycline group (Table 3). However, in MRMP, fever duration was shorter in the doxycycline group by 3 days (5 days vs. 2 days, *p* = 0.003) and days to chest X-ray improvement was shorter in the doxycycline group (6 days vs. 3 days, *p* = 0.010) (Table 3). In MRMP, fever duration was also shorter in the macrolide to doxycycline group compared with macrolide only by 3 days (5 days vs. 2 days, *p* = 0.002) and days to chest X-ray improvement (6 days vs. 3 days, *p* = 0.002) (Table 3).

**Figure 2:**
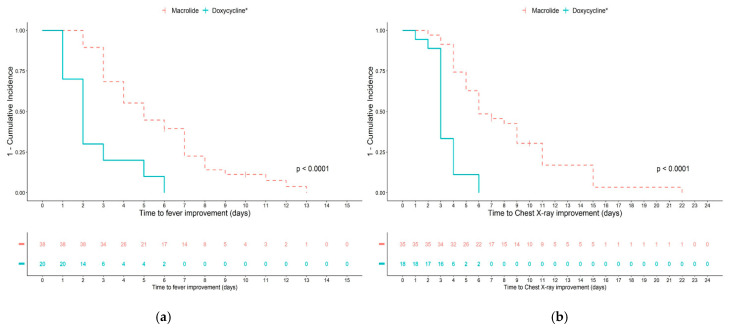
Cumulative incidence analysis of fever and chest X-ray improvement according to antibiotic group in children with macrolide-resistant *Mycoplasma pneumoniae* pneumonia. (**a**) Time to resolution of fever; (**b**) time to chest X-ray improvement in children treated with macrolides only or doxycycline (* including macrolides changed to doxycycline and doxycycline only).

The response to antibiotics among children with MRMP pneumonia at different time points was also analyzed (Table 4, Figure 2). In children infected with MRMP, more children showed defervescence in the doxycycline treated groups at 2, 3, 4, and 5 days after antibiotic treatment. When analyzing the response of chest X-ray improvement, there was no difference at 2 days; however, at day 3–5 after antibiotic treatment, a significantly higher proportion of children showed improvement in the doxycycline treated groups.

## 3. Discussion

Macrolide resistance in MP pneumonia in children has been an increasing conundrum due to limited alternative treatment options. In this study, we evaluated the clinical response to macrolides and doxycycline among children in macrolide-susceptible and macrolide-resistant cases. Among MSMP cases, there was no significant difference in clinical efficacy between macrolides and doxycycline when assessed by duration of fever or days to chest X-ray improvement after initiation of antibiotics. However, when comparing the response in MRMP, time to defervescence was reduced by 3 days and chest X-ray improvement was seen 3 days earlier in children treated with doxycycline compared to macrolides. Thus, cases with macrolide resistance showed significantly more rapid improvement of symptoms and radiologic findings with doxycycline compared with macrolides.

Doxycycline was used initially in a small number of cases among children of older ages in the 2019–2020 cohort based on the consideration of the high local macrolide resistance rate of up to 84.5%; there have been reports on the efficacy and safety of doxycycline in children and current recommendations are that children with moderate to severe disease may benefit from macrolides or tetracyclines (for children >7 years) [1,7,9]. The majority of cases which switched from macrolide to doxycycline were also in 2019–2020 (Table S1). The trend for prescription of doxycycline was related to an update in treatment guidelines for MP pneumonia in children in Korea in 2019, which recommends doxycycline as a second line therapy [10]. On the other hand, although over 40% of the subjects in this study were macrolide resistant, only 20% were treated with doxycycline as the final drug. This presumably attributes to the reluctancy of use of doxycycline in children. Taken together, macrolides stand as the first-line therapy in children; however, the difference between the two drugs in time to defervescence and chest X-ray improvement among cases with MRMP supports the benefits for switch to doxycycline in a timely manner.

As for the clinical characteristics of children with MSMP and MRMP, we found that age, sex, chest radiologic findings, viral co-infection, and need for oxygen therapy were not influenced by macrolide resistance. Additionally, there were no differences in laboratory findings, including anemia, leukocytosis, thrombocytopenia, LFT elevation, or CRP elevation (data not shown). These results correlate with previous reports, including a systemic review on the effect of macrolide resistance on clinical features in children [9,11,12,13,14]. Therefore, it is difficult to distinguish MSMP and MRMP cases based on clinical characteristics. 

When comparing the subjects who changed antibiotics after initiation of macrolides according to macrolide resistance, the proportion was higher in the MRMP at 22.4% compared with MSMP, which had a proportion of 2.5%, which was previously reported in other studies [9,12,15,16,17,18]. For optimal treatment, it would be ideal to test macrolide resistance before treatment for MP pneumonia; however, this may not always be feasible. In this study, among children treated with macrolides, we found that 68.4% of children with MRMP had fever >72 h, whereas only one-third of MSMP had persistent fever. These results are similar to previous studies on macrolide resistance [9,12,16,19,20,21]. Thus, in selected cases with persistent fever or limited evidence of radiologic improvement after 3 days of macrolide treatment, change to doxycycline may be considered.

In this study, we did not compare the difference between macrolides. However, the most common point mutations A2063G and A2064G in domain V 23S rRNA causes significant increase in the MIC of all macrolide drugs [9,22]. Previous studies have shown no significant difference between different drugs of the macrolide class [16,20]. Doxycycline has good activity against both macrolide-susceptible and -resistant strains (MIC90 0.125–0.5 μg/mL and 0.5 μg/mL, respectively). To date, no acquired resistance to tetracyclines have been reported in MP. However, reduced susceptibility has been selected in vitro with target mutations in the tetracycline-binding pocket of 16S rRNA such as G1193A and T968C [7,23,24]. Therefore, judicious use of doxycyclines and monitoring for resistance is important with the increase of use.

The overall macrolide resistance rate in this study was 40.7%. This is lower than recent reports in Korea, which were based on institutions in Seoul and other metropolitan cities in Korea [25]. In this study, cases from Jeju Island (an isolated remote island approximately 780 km south of Seoul) were included, in which the macrolide resistance rate was recently reported to be approximately 10.9% [26]. The resistance rate was also higher in 2019–2020 compared with 2014–2015; however, rather than an increase or change in antimicrobial resistance rate, this difference is related to the difference in proportion of samples from a district with low macrolide resistance rate. Thus, the resistance rate in this study does not reflect the macrolide resistance rate in Korea. The reason for the large difference between Jeju Island and other areas is not clear; however, Jeju Island is a remote area and there may have been little opportunities for introduction of MRMP strains. The institution has good accessibility and children with relatively low severity may be admitted, whereas children non-responsive to macrolides may be more likely to be admitted to institutions in the metropolitan area.

Interestingly, recent reports from Japan have shown a decrease in macrolide resistance rate of children with MP pneumonia with a peak of 81.6% in 2012 followed by 59.3% in 2014 and 43.6% in 2015 [27]. On the other hand, a report from the US among respiratory specimens collected from hospitals in eight states during 2015 and 2018 showed MRMP in 7.5% of the samples [28]. MRMP prevalence in the south and east was higher at 18.3% compared to 2.1% in the west of the US. The wide variation in macrolide resistance between time and area emphasizes the need for continuous surveillance on the macrolide resistance rate in the community.

Use of tetracyclines in children have been limited due to reports of permanent dental discoloration in children under 8 years of age. However, recent data suggest that doxycycline is not likely to cause visible teeth staining or enamel hypoplasia in young children [2]. Based on this, the American Academy of Pediatrics state that doxycycline can be administered for short durations (21 days or less) regardless of age, yet patients should be careful to avoid excess sun exposure due to photosensitivity associated with doxycycline [29]. Although, doxycycline is not considered first-line therapy for MP pneumonia in children; in this context, it may be used in selected conditions in MRMP or in areas with high prevalence of MRMP.

This study has limitations. We were not able to assess the clinical efficacy of fluoroquinolones, another option for second line therapy in MRMP, due to limited number of subjects prescribed. Additionally, there was an uneven distribution among cases in the 2014–2015 season and 2019–2020 season, which was related to the COVID-19 pandemic. Since January 2020, in response to various public health policies to control the spread of COVID-19 in the pandemic, there was a substantial decrease in respiratory infections in Korea and many resources were focused on diagnosis and management of COVID-19 [30]. However, the focus of this study was to evaluate the response to antibiotics, and all patients were grouped according to macrolide susceptibility or antibiotic regimen rather than season of infection; therefore, the difference in number of cases between seasons would not affect the results of the analysis. Additionally, this study was not designed to assess safety issues of doxycycline. Gastrointestinal symptoms, photosensitivity, and, rarely, Steven Johnson Syndrome have been reported as adverse reactions with doxycycline; however, in the limited data collection, we did not experience any cases with the severe complications necessary for discontinuance before clinical improvement.

Regardless of these limitations, in this comprehensive study, we analyzed the macrolide resistance in children with MP pneumonia and compared the clinical responses against macrolides and doxycycline based on the duration of fever and days to radiological improvement. Among children with MSMP pneumonia, there was no difference in time to improvement between macrolides and doxycycline. However, among children with MRMP pneumonia, the duration of fever and days to chest X-ray improvement was significantly shorter in cases treated with doxycycline. 

## 4. Materials and Methods

### 4.1. Study Design

We analyzed children under 18 years of age with MP pneumonia during the two recent epidemic seasons in 2014–2015 and 2019–2020. Four hospitals participated in this study, including Seoul National University Children’s Hospital (Seoul), Seoul National University Bundang Hospital (Seongnam), Chungnam University Hospital (Daejeon), and Jeju University Hospital (Jeju Island).

To compare the response between the antibiotics, the primary outcome was days to defervescence, and secondary outcome was days to improvement in chest radiography, defined as more than 30% improvement.

The inclusion criteria were the following: (1) children with febrile pneumonia, (2) treated with macrolides or doxycycline, (3) serologic evidence for MP infection [serum anti-mycoplasma IgM positive, single anti-mycoplasma IgG titer ≥ 1:640 or paired anti-mycoplasma IgG titer show 4-fold increase] and [culture or polymerase chain reaction (PCR) positive for MP], and (4) tested for macrolide resistance [31]. Anti-mycoplasma IgM and IgG were tested using ELISA (Seegene, Seoul Korea) and anti-mycoplasma IgG was titrated using an indirect particle agglutination test kit (SerodiaMycoII, Fujirebio, Tokyo, Japan) according to the manufacturer’s instructions. Pneumonia was defined with the presence of infiltration in chest radiography with clinical symptoms and signs such as fever (defined as ≥38 °C), cough, or sputum [11]. Children with underlying diseases, including chronic lung disease or immunodeficiency, bacteria co-infection such as *Streptococcus pneumoniae*, *Staphylococcus aureus* or *Bordetella pertussis*, treatment with steroids, children for whom data were not available for defervescence, and children lost to follow-up were excluded from the study. Additionally, to assess the response to antibiotics, children treated for at least 5 days or more were included.

Nasopharyngeal aspirates or swabs were obtained from children with acute lower respiratory tract infections as a strategy of routine patient care at admission. Multiplex PCR was performed to detect respiratory viruses, including respiratory syncytial virus, influenza virus, parainfluenza virus, adenovirus, rhinovirus, and coronavirus (Seeplex RV12 ACE detection kit, Seegene, Seoul, Korea). Residual samples were sent to the laboratory of the Seoul National University Children’s Hospital for the culture and PCR of MP and analysis of macrolide resistance. Macrolide resistance was analyzed retrospectively and results were not available during treatment. 

### 4.2. Detection of Macrolide Resistance of MP

Culture and PCR of MP was performed as previously described [11]. Macrolide resistance was determined by the presence of gene transitions on the domain V of the 23S rRNA gene. Briefly, to amplify domain V of the 23S rRNA gene, PCR analysis was performed on cultured MP isolates or DNA extracted from nasopharyngeal samples. The primers used were MP23SV-F (5′-TAA CTA TAA CGG TCC TAA GG) and MP23SV-R (5′-ACA CTT AGA TGC TTT CAG CG). DNA from the reference strain M129 (ATCC 29342) was used as a positive control, and distilled water was used as a negative control; amplifications of samples were performed for 35 cycles. The 851-bp PCR products were purified using an AccuPrep® PCR Purification Kit (Bioneer, Inc., Daejeon, Korea), and samples were sequenced to identify the transitions in domain V macrolide resistance, such as A2063G and A2064G [32]. The minimal inhibitory concentration for macrolides in strains with 23S rRNA transition were previously reported [9]. Based on these results, cases were classified as MSMP or MRMP.

### 4.3. Clinical Data Collection and Assessment of Antimicrobial Response

The electronic medical records of all patients were reviewed. Data collected included demographic factors, clinical manifestations, laboratory results, antimicrobial agents, concomitant medication, and outcome including duration of symptoms. The duration of fever was defined as the number of days for which the patient had a body temperature of ≥38 °C with an interval of <24 h between each episode of fever. Medical records were reviewed by a physician in each hospital and collected on a standardized from.

Chest X-ray findings were categorized into homogeneous dense lobar consolidation, patchy consolidation, nodular opacities, and perihilar peribronchial infiltration [11]. Parapneumonic effusion, defined as ≥1 cm width on the decubitus view, was also included in the analysis. In the four participating hospitals, all physicians were unaware of the results of 23S rRNA mutation during the treatment.

According to treatment, children were classified into macrolide, doxycycline, and macrolide to doxycycline group. For evaluation of antimicrobial response, the days to defervescence and days to chest X-ray improvement was evaluated. 

### 4.4. Statistical Analyses

All statistical analyses were performed with R version 4.0.1. (R Foundation for statistical computing, Vienna, Austria). Differences between categorical variables were tested using the Chi-squared or Fisher’s exact test. The Wilcoxon Rank Sum or Kruskal-Wallis test were used to compare the age, days to defervescence, and days to chest X-ray improvement between the treatment groups as appropriate. In all analyses, a *p*-value of ≤0.05 was considered statistically significant.

## 5. Conclusions

Doxycycline is effective and may be considered in selected cases in children with MRMP pneumonia. 

## Figures and Tables

**Table 1:** Demographics and clinical characteristics of children with *Mycoplasma pneumoniae* pneumonia according to macrolide resistance.

Demographics and Clinical Characteristics	Total (*n* = 145)	MSMP (*n* = 86)	MRMP (*n* = 59)	*p* Value *
Age, years, median (IQR)	5 (4, 8)	5 (3, 8)	6 (4, 9)	0.175
Male gender	59 (40.7)	33 (38.4)	26 (44.1)	0.498
Underlying disease	8 (5.5)	3 (3.5)	5 (8.5)	0.160
Diagnostics ^†^				
*M. pneumoniae* PCR ^‡^	127/138 (92.0)	83/86 (95.5)	44/52 (84.6)	0.020
*M. pneumoniae* culture ^§^	66/73 (90.4)	19/22 (86.4)	47/51 (92.2)	0.048
Antimycoplasma IgM	32/51 (62.7)	22/37 (59.5)	10/14 (71.4)	0.527
Antimycoplasma IgG ≥ 1:640	57/118 (48.3)	36/75 (48.0)	21/43 (43.8)	1.000
Antimycoplasma IgG 4-fold increase	35/37 (94.6)	19/19 (100)	16/18 (88.9)	0.230
Fever, days, median (IQR)	7 (6, 9)	7 (5, 9)	8 (7, 11)	0.003
Cough, days, median (IQR)	15 (11, 18.25)	14 (10, 18)	16 (12, 19)	0.249
Radiologic findings				
Perihilar peribronchial infiltration	28 (19.4)	17 (19.7)	11 (18.7)	0.992
Nodular	19 (13.1)	11 (12.8)	8 (13.6)	
Patchy consolidation	40 (27.6)	23 (26.7)	17 (28.8)	
Lobar consolidation	58 (40.0)	35 (40.7)	23 (39.0)	
Parapneumonic effusion	16 (11.0)	10 (11.6)	6 (10.2)	
Viral co-infection ^¶^	18 (12.4)	11 (12.8)	7 (11.9)	0.998
Outcome				
Improved without complication	143 (98.8)	86 (100.0)	57 (96.6)	0.161
Improved with complication	2 (1.4)	0 (0)	2 (3.4)	
Institution				
A	54 (37.2)	14 (16.3)	40 (67.8)	<0.001
B	11 (7.6)	4 (4.7)	7 (11.9)	
C	6 (4.1)	2 (2.3)	4 (6.8)	
D	74 (51.0)	66 (76.7)	8 (13.6)	
Year (season)				
2014–2015	103 (71.0)	72 (69.9)	31 (30.1)	<0.001
2019–2020	42 (29.0)	14 (33.3)	28 (66.7)	

MSMP, macrolide-susceptible *Mycoplasma pneumoniae*; MRMP, macrolide-resistant *Mycoplasma pneumoniae,* * MSMP vs. MRMP, Data are no. (%) of patients unless otherwise indicated, ^†^ Data are no. positive/no. tested (%), ^‡^ Samples shown are results in each institution laboratory, ^§^ Institute D only performed PCR in 2014–2015, ^¶^ Viral co-infection (Respiratory syncytial virus, RSV *n* = 3, Adenovirus *n* = 3, Parainfluenza virus *n* = 3, Rhinovirus *n* = 1, Influenza *n =* 1, Enterovirus *n* = 1, Human metapneumovirus *n* = 1, Adenovirus and RSV *n* = 2, Adenovirus and Parainfluenza virus *n* = 1, Adenovirus and Influenza *n* = 1, Coronavirus and Rhinovirus *n* = 1).

**Table 2:** Antibiotic treatment in of children with *Mycoplasma pneumoniae* pneumonia according to macrolide resistance.

Antibiotic	Total (*n* = 145)	MSMP (*n* = 86)	MRMP (*n* = 59)	*p* Value *
**Antibiotic regimen**				<0.001
Macrolide	116 (80.0)	78 (90.7)	38 (64.6)	
Macrolide to Doxycycline	13 (9.0)	2 (2.3)	11 (18.6)	
Doxycycline only	16 (11.0)	6 (7.0)	10 (16.9)	
**Final antibiotic**				<0.001
Macrolide	116 (80.0)	78 (90.7)	38 (64.6)	
Doxycycline ^†^	29 (20.0)	8 (9.3)	21 (35.6)	

MSMP, macrolide-susceptible *Mycoplasma pneumoniae*; MRMP, macrolide-resistant *Mycoplasma pneumoniae*; * Macrolide-susceptible vs. Macrolide-resistant, ^†^ Includes macrolide changed to doxycycline and doxycycline only. Data are no. (%) of patients.

**Table 3:** Clinical response according to antibiotic treatment in children with *Mycoplasma pneumoniae* pneumonia related to macrolide resistance.

Parameter	Macrolide Only		Macrolide to Doxycycline		Doxycycline Only		*p* Value *
	*n*	Duration	*n*	Duration	*n*	Duration	
Fever							
MSMP	78	3.0 (3.0, 5.0)	2	2 (2, 2)	6	2.5 (1.25, 3.0)	0.048
MRMP	38	5.0 (3.0, 7.0)	11	2 (1.5, 2.5)	10	2.0 (1.25, 2.75)	<0.001
*p* value ^†^		0.0005		>0.999		0.9106	
Chest X-ray improvement							
MSMP	72	4.5 (4.0, 7.0)	2	4.5 (3.8, 5.3)	5	4.0 (4.0, 5.0)	0.890
MRMP	35	6.0 (4.5, 10.5)	9	3 (3.0, 4.0)	9	3.0 (3.0, 4.0)	<0.001
*p* value ^†^		0.0482		0.3821		0.2499	

MSMP, macrolide-susceptible *Mycoplasma pneumoniae*; MRMP, macrolide-resistant *Mycoplasma pneumoniae,* * Comparison between three groups, ^†^ MSMP vs. MRMP; Data are median (IQR); Duration of fever was defined as days of fever after initiation of antibiotic; Chest X-ray improvement was defined as more than 30% improvement.

**Table 4:** Cumulative incidence of fever or chest X-ray improvement after antibiotic initiation in macrolide-resistant *Mycoplasma pneumoniae* pneumonia.

Parameter	Time Point	All	Macrolide	Doxycycline *	*p* Value
Resolution of fever	Total	58	38	20	
	48 h	18 (31.0)	4 (10.5)	14 (70.0)	<0.001
	72 h	28 (48.3)	12 (31.6)	16 (80.0)	0.001
	96 h	33 (56.9)	17 (44.7)	16 (80.0)	0.013
	120 h	39 (67.2)	21 (55.3)	18 (90.0)	0.008
Chest X-ray improvement	Total	53	35	18	
	48 h	3 (5.7)	1 (2.9)	2 (11.1)	0.263
	72 h	15 (28.3)	3 (8.6)	12 (66.7)	<0.001
	96 h	25 (47.2)	9 (25.7)	16 (88.9)	<0.001
	120 h	29 (54.7)	13 (37.1)	16 (88.9)	<0.001

* Includes macrolide changed to doxycycline and doxycycline only, Data are no. (%) of patients.

## Data Availability

The data presented in this study are available on request from the corresponding author.

## Supplementary Materials

The following are available online at https://www.mdpi.com/2079-6382/10/2/192/s1. Table S1: Demographics and clinical characteristics of children with *Mycoplasma pneumoniae* pneumonia according to antibiotic treatment.
