# Supplementary material for: Clinical Efficacy of Doxycycline for Treatment of Macrolide-Resistant Mycoplasma pneumoniae Pneumonia in Children
Source: Antibiotics (Basel). 2021 Feb 17;10(2):192. doi: 10.3390/antibiotics10020192 (PMC7921960; doi:10.3390/antibiotics10020192)
Supplement: Supplementary file 1 [file antibiotics-10-00192-s001.pdf]

Table 1S. Demographics and clinical characteristics of children with *Mycoplasma pneumoniae* pneumonia according to antibiotic treatment

	Total (N=145)	Macrolide only (N=116)	Macrolide to Doxycycline (N=13)	Doxycycline only (N=16)	*P* value*
Age, years, median (IQR)	5 (4, 8)	5 (4, 8)	9 (7, 9)	8 (6, 10.25)	0.001
Male gender	59 (40.7)	47 (40.5)	3 (23.1)	9 (56.2)	0.218
Macrolide resistance	59 (40.7)	38 (32.8)	11 (84.6)	10 (62.5)	<0.001
Fever, days, median (IQR)	7 (6, 9)	7 (5, 9)	9 (7, 11)	7 (6, 8.25)	0.461
Cough, days, median (IQR)	15 (11, 18.25)	114 (98.3)	12 (92.3)	16 (100.0)	0.276
Radiologic findings					0.263
Perihilar peribronchial infiltration	28 (19.4)	25 (21.6)	2 (15.4)	1 (6.2)	
Nodular	19 (13.1)	16 (13.8)	1 (7.7)	2 (12.5)	
Patchy consolidation	40 (27.6)	27 (23.3)	5 (38.5)	8 (50.0)	
Lobar consolidation	58 (40.0)	48 (41.4)	5 (38.5)	5 (31.2)	
Parapneumonic effusion	16 (11.0)	13 (11.2)	1 (7.7)	2 (12.5)	
Viral co-infection†	18 (12.4)	13 (11.2)	2 (15.4)	3 (18.8)	0.429
Result					
Improved	143 (98.8)	114 (98.3)	13 (100.0)	16 (100.0)	1.000
Complication	2 (1.4)	2 (1.7)	0 (0.0)	0 (0.0)	
Institution					
A	54 (37.2)	33 (38.4)	7 (53.8)	14 (87.5)	<0.001
B	11 (7.6)	9 (7.8)	2 (15.4)	0 (0.0)	
C	6 (4.1)	2 (1.7)	2 (15.4)	2 (12.5)	
D	74 (51.0)	72 (62.1)	2 (15.4)	0 (0.0)	
Year (season)					
2014-2015	103 (71.0)	101 (87.1)	2 (15.4)	0 (0.0)	<0.001
2019-2020	42 (29.0)	15 (12.9)	11 (84.6)	16 (100.0)	

*Macrolide only vs. Macrolide to Doxycycline vs. Doxycycline only, Data are no. (%) of patients unless otherwise indicated, † Viral co-infection (RSV N=3, Adenovirus N=3, Parainfluenza virus N=3, Rhinovirus N=1, Influenza N=1, Enterovirus N=1, Human metapneumovirus N=1, Adenovirus & RSV N=2, Adenovirus & Parainfluenza virus N-1, Adenovirus + Influenza N=1, Coronavirus+Rhinovirus N=1)
